# Experimental Demyelination and Axonal Loss Are Reduced in MicroRNA-146a Deficient Mice

**DOI:** 10.3389/fimmu.2018.00490

**Published:** 2018-03-12

**Authors:** Nellie A. Martin, Viktor Molnar, Gabor T. Szilagyi, Maria L. Elkjaer, Arkadiusz Nawrocki, Justyna Okarmus, Agnieszka Wlodarczyk, Eva K. Thygesen, Miklos Palkovits, Ferenc Gallyas, Martin R. Larsen, Hans Lassmann, Eirikur Benedikz, Trevor Owens, Asa F. Svenningsen, Zsolt Illes

**Affiliations:** ^1^Department of Neurology, Odense University Hospital, Odense, Denmark; ^2^Department of Genetics, Cell- and Immunobiology, Semmelweis University, Budapest, Hungary; ^3^Department of Biochemistry and Clinical Chemistry, University of Pécs, Pécs, Hungary; ^4^Department of Biochemistry and Molecular Biology, University of Southern Denmark, Odense, Denmark; ^5^Department of Neurobiology Research, Institute for Molecular Medicine, University of Southern Denmark, Odense, Denmark; ^6^Laboratory of Neuromorphology and Human Brain Tissue Bank, Microdissection Laboratory, Semmelweis University, Budapest, Hungary; ^7^Szentagothai Research Centre, University of Pécs, Pécs, Hungary; ^8^Nuclear-Mitochondrial Interactions Research Group, Hungarian Academy of Sciences, Budapest, Hungary; ^9^Center for Brain Research, Medical University of Vienna, Vienna, Austria; ^10^Department of Clinical Research, University of Southern Denmark, Odense, Denmark

**Keywords:** cuprizone, miR-146a, miR-181b, miR-193a, demyelination, remyelination, multiple sclerosis

## Abstract

**Background:**

The cuprizone (CPZ) model of multiple sclerosis (MS) was used to identify microRNAs (miRNAs) related to *in vivo* de- and remyelination. We further investigated the role of miR-146a in miR-146a-deficient (KO) mice: this miRNA is differentially expressed in MS lesions and promotes differentiation of oligodendrocyte precursor cells (OPCs) during remyelination, but its role has not been examined during demyelination.

**Methods:**

MicroRNAs were examined by Agilent Mouse miRNA Microarray in the corpus callosum during CPZ-induced demyelination and remyelination. Demyelination, axonal loss, changes in number of oligodendrocytes, OPCs, and macrophages/microglia was compared by histology/immunohistochemistry between KO and WT mice. Differential expression of target genes and proteins of miR-146a was analyzed in the transcriptome (4 × 44K Agilent Whole Mouse Genome Microarray) and proteome (liquid chromatography tandem mass spectrometry) of CPZ-induced de- and remyelination in WT mice. Levels of proinflammatory molecules in the corpus callosum were compared in WT versus KO mice by Meso Scale Discovery multiplex protein analysis.

**Results:**

miR-146a was increasingly upregulated during CPZ-induced de- and remyelination. The absence of miR-146a in KO mice protected against demyelination, axonal loss, body weight loss, and atrophy of thymus and spleen. The number of CNP^+^ oligodendrocytes was increased during demyelination in the miR-146a KO mice, while there was a trend of increased number of NG2^+^ OPCs in the WT mice. miR-146a target genes, SNAP25 and SMAD4, were downregulated in the proteome of demyelinating corpus callosum in WT mice. Higher levels of SNAP25 were measured by ELISA in the corpus callosum of miR-146a KO mice, but there was no difference between KO and WT mice during demyelination. Multiplex protein analysis of the corpus callosum lysate revealed upregulated TNF-RI, TNF-RII, and CCL2 in the WT mice in contrast to KO mice. The number of Mac3^+^ and Iba1^+^ macrophages/microglia was reduced in the demyelinating corpus callosum of the KO mice.

**Conclusion:**

During demyelination, absence of miR-146a reduced inflammatory responses, demyelination, axonal loss, the number of infiltrating macrophages, and increased the number of myelinating oligodendrocytes. The number of OPCs was slightly higher in the WT mice during remyelination, indicating a complex role of miR-146a during *in vivo* de- and remyelination.

## Introduction

Multiple sclerosis (MS) is an inflammatory demyelinating disease of the central nervous system [CNS; (1)]. Immunomodulatory treatments are available for prevention of relapses in the relapsing–remitting form of the disease, but treatment options to prevent demyelination are limited (2).

It has been estimated that more than 60% of all protein-coding mammalian genes can be regulated by microRNAs (miRNA) (3). The functional level of miRNAs can be manipulated *in vivo* (4), and clinical trials are already running with the intention of either restoring miRNA function by administration of miRNA mimics (5) or inhibiting their function by antimiR oligonucleotids (6). The posttranscriptional regulatory system of microRNAs has been found to be extensively involved in almost all biological processes, including those essential in the pathology of MS (7). Many microRNAs are differentially regulated in response to MS in brain lesions (8, 9), whole blood (10, 11), isolated blood cells (12, 13), plasma and serum (14, 15), and cerebral spinal fluid (16, 17). Still, the actual role of these differentially expressed miRNAs in MS pathology has been not extensively explored. One of the miRNAs that is differentially regulated in different tissues and cells in MS is miR-146a: it has been found to be upregulated in brain lesions (8), serum (18) and blood-derived immune cells (13, 19). The role of miR-146a as a negative regulator of immune activation is well established (20, 21). miR-146a is involved in a negative feedback loop: it is induced by NF-kB, but also inhibits the activation of NF-κB (22). miR-146a is involved in cell death and survival; in glioma cells: overexpression of miR-146a suppressed cell survival, proliferation, and migration, whereas inhibition resulted in improved migration potential (23, 24). miR-146a amplified the effect of a G-actin-sequestering peptide to promote OPC differentiation *in vitro*, and its overexpression in neural progenitor cells increased their differentiation into OPCs (25, 26). Recently, infusion of miR-146a mimics into demyelinated corpus callosum after 5-week administration of cuprizone (CPZ) promoted differentiation of OPCs into myelinating oligodendrocytes (27).

Oral administration of the copper chelator bis-cyclohexanone-oxalyldihydrazone (CPZ) leads to demyelination most pronounced in the corpus callosum, whereas discontinuation of CPZ results in rapid remyelination (28). The mechanism of CPZ-induced demyelination is not fully understood but mitochondrial dysfunction and oxidative stress to which oligodendrocytes are particularly sensitive is suspected (29, 30). The blood–brain barrier remains intact, and the absence of T and B cells do not affect CPZ-induced demyelination (31). Therefore, de- and remyelination can be examined as relatively separated processes unmasked from the contribution of adaptive immune responses, in contrast to another commonly used MS model, experimental autoimmune encephalomyelitis (EAE) (28, 30).

Considering the differential expression of microRNAs in different tissues and compartments of patients with MS, here we used the CPZ mouse model of MS to identify and investigate miRNAs involved in de- and remyelination. By using microarray, we identified three miRNAs differentially expressed during experimental de- and remyelination that were also reported to be differentially expressed in MS lesions (8). One of these miRNAs, miR-146a can have both pro- anti-apoptotic effect in different cells, including OPCs (26, 32), and had a unique expression profile during de- and remyelination in our experiments. This miRNA has been recently investigated in the CPZ model, and its injection promoted differentiation of OPCs into myelinating oligodendrocytes during remyelination in wild-type (WT) mice (27). Here, we investigated, if the absence of miR-146a influences demyelination in the CPZ model by using miR-146a-deficient (KO) mice.

## Materials and Methods

### miR-146a KO Mice

miR-146a knockout (KO) mice were purchased from the Jackson Laboratory (ME, USA). This mouse strain was generated on a C57BL/6 background in Dr. David Baltimore’s laboratory, California Institute of Technology (33). Mice were bred at the Biomedical Laboratory, SDU according to protocols and guidelines approved by the Danish Animal Health Care Committee (2014-15-00369). All animal experiments complied with the EU Directive 2010/63/EU for animal experiments. Female KO mice aged 7–8 weeks were included in the experiments. At this age, miR-146a KO mice do not display a visible autoimmune or inflammatory phenotype (33).

### Cuprizone-Induced Demyelination and Remyelination

Cuprizone (Sigma Aldrich, MO, USA) was delivered orally: powdered standard chow was mixed with 0.2–0.4% CPZ. To induce demyelination, CPZ was administrated to 7- to 8-week-old mice for 4 weeks (4 weeks demyelination: 4wd). Remyelination was examined at two time-points: acute remyelination induced by 4 weeks CPZ feeding followed by 2 days of regular diet (2 days remyelination: 2dr), and full remyelination induced by 4 weeks CPZ feeding followed by 2 weeks of regular diet (2 weeks remyelination: 2wr). Control mice were kept on a normal diet. During experiments, mice were weighed every second day to control that mice lost no more than maximum 20% of their body weight. Experiments were terminated by euthanizing mice with an overdose of pentobarbital (Glostrup Apotek, Glostrup, Denmark) followed by perfusion with 4% paraformaldehyde (PFA) for staining applications, or phosphate buffered saline for all other experiments.

### Extraction of Whole RNA and Quantitative PCR (qPCR)

For the removal of the corpus callosum, the brains were removed from the skull, immediately frozen, and cut into coronal serial sections (section thickness 200 µm). By using a stereomicroscope, the corpus callosum was cut out of the sections with a fine Graefe-knife, along its rostro-caudal extension. The samples were collected in ice-cold Eppendorf-tubes and stored frozen until used. RNA was extracted by the miRNeasy micro Kit (Qiagen, Valencia, CA, USA). The quantity and quality of total RNA was assessed by NanoDrop ND-1000 spectrophotometer (NanoDrop Technologies, Wilmington, DE, USA) and Agilent 2100 Bioanalyzer (Agilent Technologies, Palo Alto, CA, USA), respectively. Only those samples were used for microarray experiments that gave >8.0 for RNA integrity number, showed a clear gel image, and no DNA contamination was observed on the histogram.

To measure miRNA expression, primer sets for specific miRNA assays and sno135 endogenous control and the MicroRNA Reverse Transcription Kit (Life Technologies, Thermo Scientific, CA, USA) were utilized following the manufacturer’s protocol. Briefly, 10 ng of each total RNA sample were transcribed by MultiScribe Reverse Transcriptase. qPCR was carried out using Applied Biosystems 7000 Real-Time PCR System. Specific TaqMan chemistry primers for reverse transcription and qPCR were acquired from Life Technologies (Thermo Scientific) (miR-146a ID: 000468, snoRNA135 ID: 001230, CA, USA). Reverse transcription was conducted under the following conditions: 30 min at 16°C, 30 min at 42°C, 5 min at 85°C, cool down to 4°C; and for qPCR analyses the following conditions were applied: 2 min at 50°C, 10 min at 95°C, 40 cycles of 15 s at 95°C followed by 1 min at 60°C according the manufacturer’s instructions. The relative expression of each miRNA was calculated from the equation 2-ΔCt, where ΔCt = mean Ct(miRNA)—mean Ct(internal control) (where Ct is the threshold cycle for a sample). All reagents and instruments for qPCR were purchased from Applied Biosystems Inc. except when otherwise indicated.

### miRNA Microarray

For microRNA profiling, the Agilent Mouse miRNA Microarray Kit (G4472A, 8 × 15k) was applied according to the manufacturer’s instruction (version 1.0) with 100–100 ng quality-checked total RNA. The labeled samples were hybridized for 20 h at 55°C. The arrays were scanned with an Agilent DNA Microarray Scanner BA, the signal quantification was carried out by Feature Extraction 10.7 Image Analysis Software and data were further analyzed by Genespring GX10.0. The microarray data are deposited in NCBI Gene Expression Omnibus with accession GSE100662.

### Meso Scale Discovery Multiplex Electrochemiluminescent Assay

Cytokine levels in the corpus callosum were measured by the Meso Scale Discovery (MSD, USA) electrochemiluminescence proinflammatory mouse V-Plex Plus Kit (IL-1β, IL-4, IL-6, IL-10, TNF), a MULTI-SPOT 4 spot cytokine costume plate (MIP1α, VEGF, and MMP9) and a MULTI-SPOT 2 spot cytokine costume plate (TNF-RI and TNF-RII). We used a SECTOR Imager 6000 (Meso Scale Discovery) Plate Reader, and data were analyzed using the MSD Discovery Workbench software according to the manufacturer’s instructions. Results are presented relative to the total protein concentration of the individual samples.

### ELISA

SMAD4 and SNAP25 protein levels in the corpus callosum were examined and compared between miR-146a KO mice and WT by ready-made Sandwich ELISA kits according to the manufacturer’s instructions (Nordic Biosite, OKEH03425 and EKM1284, respectively). A Molecular Devices, Vmax kinetic microplate reader was used to analyze the results, and results are presented relative to the total protein concentration of the individual samples.

### Histopathology

Brains were postfixed in 4% PFA overnight before they were embedded in paraffin. Then, 8 µm coronal sections were obtained at the levels of 161, 181, 209, and 221 (34). Demyelination was evaluated using Luxol fast blue staining with cresyl violet. Axonal pathology was examined by Bielschowsky staining. The sections in the corpus callosum were overlaid by a 100-point grid at a magnification of 20× in the microscope and the number of points, each representing 2.5 mm^2^, located within the lesion was counted. First, the size of the entire lesion was determined (total lesion). Next, the size of the area, which showed remyelination (evenly thin myelin sheaths) was measured. Immunocytochemistry was performed on paraffin sections as described before (35) without antigen retrieval using antibodies against Iba1 (WAKO #019-19741), Mac3 (Becton & Dickinson #553322), NG2 (Millipore AB 5320), and CNP (Sternberger Monoclonals SMI 91). Stained cells were counted in sections overlaid with a morphometric grid in the ocular lens. The cell count values represent cells/mm^2^.

### Proteomics

The proteomic analysis was performed in pentaplicate. The corpus callosum was dissected from fresh-frozen brain obtained from mice during demyelination, acute remyelination (2 days), full remyelination (2 weeks), and controls (five mice in each group). The samples were treated with protease and phosphatase inhibitors, ultracentrifuged to remove cell debris and nano-structures and subsequently the total protein content was estimated by amino acid composition analysis. The proteins in the supernatant were reduced with DTT, alkylated with iodoacetamide and digested with trypsin as described elsewhere (36). The purified peptides from each pool were labeled with one of the iTRAQ reagent according to the manufactures protocol and respective labeled samples were mixed in 1:1 proportion (based on the amino acids composition analysis). In order to enrich for phosphopeptides and glycosylated peptides, the TiSH protocol was applied (37). Peptide samples were fractioned and analyzed by LC-MS/MS using a nano-Easy LC (Thermo Fisher Scientific) coupled with either a Q-Exactive or Velos mass spectrometers (Thermo Fisher Scientific, Bremen, Germany). All peptides fractions were resuspended in 0.1% FA and loaded onto a 2-cm 100 µm inner diameter pre-column using the nano-Easy LC. Peptides were eluted directly onto the analytical column using a gradient of 0–34% buffer B (90% Acetonitrile, 0.1% FA) over 30–120 min depending on the UV intensity of the individual HILIC fractions. All LC-MS/MS runs were performed using an analytical column of 20 cm × 75 µm inner diameter fused silica, packed with C18 material (Dr. Maisch, Ammerbuch-Entringen, Germany). The MS settings for Q-Exactive instrument were as follows: full MS: resolution at 60,000, AGC target 1e6, Maximum IT 100 ms, and for data-dependent MS/MS of the top 12 most intense ions: resolution at 15,000, AGC target 2e4, Maximum IT 100 ms, isolation window 1.2 *m/z*, fixed first mass 110.0 *m/z*, NCE: 30, Intensity threshold 1e4. For the analysis on Velos instrument, the settings were similar, but top 7 most intense ions were selected for MS/MS fragmentation at 7,500 resolution, NCE 35. The MS raw files were processed and search in Mascot and SEQUEST through the Proteome Discoverer 2.1 software (Thermo Fisher Scientific). Database searches were performed using the following parameters: Precursor mass tolerance of 10 parts per million (ppm); MSMS mass tolerance of 0.05 Da; Enzyme: trypsin and up to two missed cleavages were allowed.

### Experimental Design and Statistical Analysis

If not otherwise stated, statistical tests were performed using Prism 7 software (GraphPath, USA, CA, USA) and quantitative data are presented as mean ± SEM. Exact *p*-values are specified for all ANOVA tests when *p* > 0.0001, and *p* < 0.05 is considered significant. Each ANOVA test is followed by an appropriate *post hoc* test.

Raw miRNA expression data were obtained from 3 to 4 mice pr. group. The microarray data were normalized to the 75th percentile signal intensity and entities showing present call in all samples of a condition were filtered out. Differentially expressed genes were selected when passing the signal intensity filter (entities where at least 100% of samples in any one out of four conditions have values within cutoff) and showing at least twofold statistically significant change (ANOVA and Tukey HSD *post hoc* test, with Benjamini–Hochberg multiple testing correction *p*-value <0.05) between any of the groups. Validation of miRNA expression by qPCR included five to eight mice in each group and data were analyzed by one-way ANOVA followed by LSD *post hoc* tests. Body weight, thymus and spleen weight, and lesion size analysis included 4–16, 4–8, and 4–7 mice at each time-point, respectively, and data were analyzed using two-way ANOVA followed by Bonferroni *post hoc* tests.

For the analysis of the proteome, five mice were included. The ratio (*r*) for each protein, in any of the three comparisons (any condition to the control) was compared to its SE, such that if *r* ≤ 1/(1 + 2SE) or if *r* ≥ (1 + 2SE), the protein has changed. If the protein could be measured only in one or two of the five mice, they were not encountered in this analysis.

ELISA analyses of SMAD4 and SNAP25 and Meso Scale Discovery multiplex analysis of cytokines, chemokines, and TNF receptors included four to eight mice in each group, and data were analyzed using two-way ANOVA followed by Bonferroni *post hoc* tests.

## Results

### Differential Expression of MicroRNAs in the Corpus Callosum During CPZ-Induced Demyelination and Remyelination

**Figure 1:**
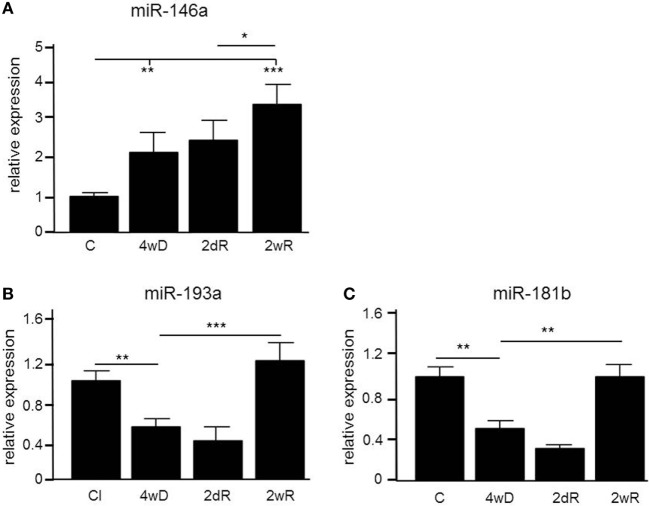
Differential expression of microRNAs (miRNAs) in the corpus callosum of mice during experimental demyelination and remyelination. Using microarray and validation by quantitative PCR (qPCR), three miRNAs, miR-146a **(A)**, miR-193a **(B)** and miR-181b **(C)** were differentially regulated in response to CPZ exposure in the corpus callosum. *p < 0.05, **p < 0.01, ***p < 0.001, *n* = 5–8 in each group, one-way ANOVA, mean ± SEM. Abbreviation: C: un-manipulated controls covering ages of mice used in the de- and remyelinating experiments, 4wD: 4 weeks demyelination. 2dR: 2 days (acute) remyelination, 2wR: 2 weeks (full) remyelination.

In order to identify microRNAs involved in the pathology of de- and remyelination, we isolated the corpus callosum from mice exposed to CPZ and conducted an Agilent microarray analysis for 627 miRNAs (data are deposited in NCBI Gene Expression Omnibus with accession GSE100662). We identified three miRNAs, miR-146a, miR-181b, and miR-193a, which were differentially expressed compared to controls confirmed by qPCR (Figure 1). The expression of miR-146a increased in response to CPZ exposure, and continued to increase during the remyelination phase (*p* < 0.001, one-way ANOVA, LSD *post hoc* test) (Figure 1A). By contrast, the expression level of miR-193a and miR-181b decreased in response to CPZ-induced demyelination and had returned to baseline in the full remyelination phase (*p* < 0.001 and *p* < 0.01, respectively, one-way ANOVA, LSD *post hoc* test) (Figures 1B,C).

Observing (i) a continuous increase in the expression level of miR-146a in contrast to the two other miRNAs, (ii) considering its biological function, and (iii) also the differential expression in brain lesions, body fluids, and cells obtained from MS patients (8, 13, 18, 19, 25–27), we further investigated the role of miR-146a in CPZ-induced de- and remyelination.

### Expression of miR-146a in Response to CPZ in Different Organs

**Figure 2:**
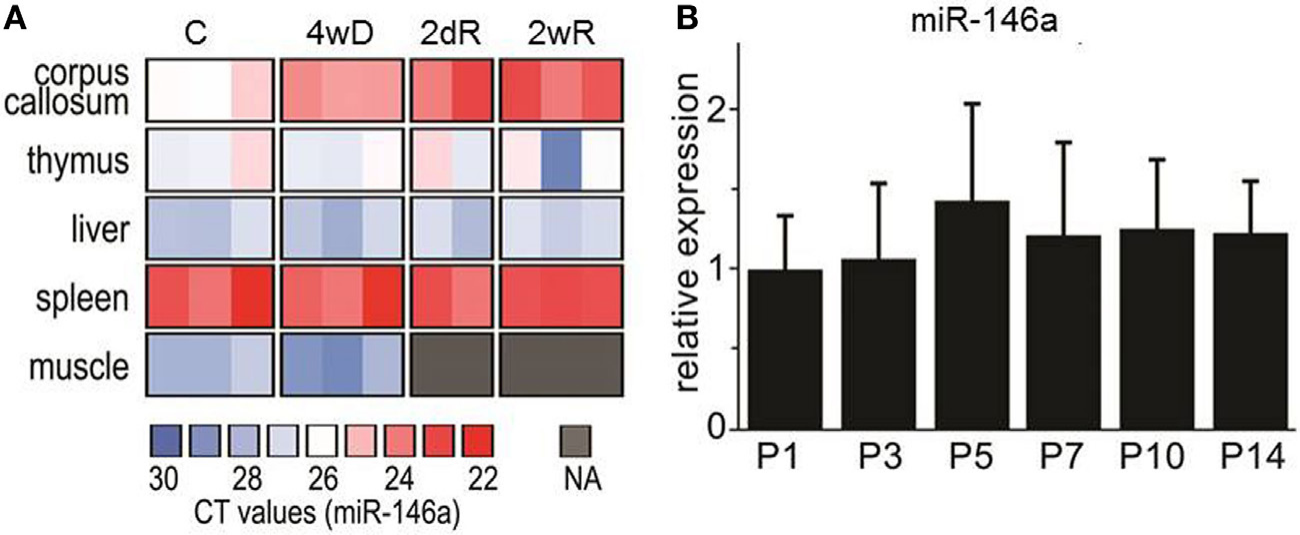
Expression of miR-146 in different organs during cuprizone (CPZ) treatment and during physiological postnatal myelination. **(A)** The relative expression of miR-146a was determined by qPCR in different organs of individual animals. Abbreviation: C: un-manipulated controls, 4wD: 4 weeks demyelination. 2dR: 2 days (acute) remyelination, 2wR: 2 weeks (full) remyelination. **(B)** The expression of miR-146a in the corpus callosum during physiological myelination (on postnatal days 1–14, P1–P14). The relative expression of miR-146a was determined by qPCR and the results are presented relative to sno135 (*n* = 4 in each group, one-way ANOVA, mean ± SEM).

In addition to analyzing the expression level of miR-146a in the corpus callosum, we also analyzed the expression level in thymus, liver, spleen, and muscle tissue. In contrast to the corpus callosum, we did not see a CPZ-induced increase of miR-146a in any of these organs. The highest expression level of miR-146a among the examined organs was found in the spleen (Figure 2A).

### Expression of miR-146a in the Brain During Physiological Myelination in Postnatal Mice

In order to investigate, if the level of miR-146a is also increased during physiological myelination, we examined its expression in the corpus callosum isolated from postnatal mice aged 1 –14 days (P1–P14); this is the most critical period for physiological myelination in mice (38). We found no change in the expression of miR-146a, which indicates that the observed increase in response to CPZ exposure is associated with demyelination pathology (Figure 2B).

To further examine the effect of miR-146 on de- and remyelination *in vivo*, we used a miR-146a-deficient (KO) strain compared to WT mice in additional experiments (33).

### Systemic Effects of CPZ Exposure in miR-146a-Deficient Mice

**Figure 3:**
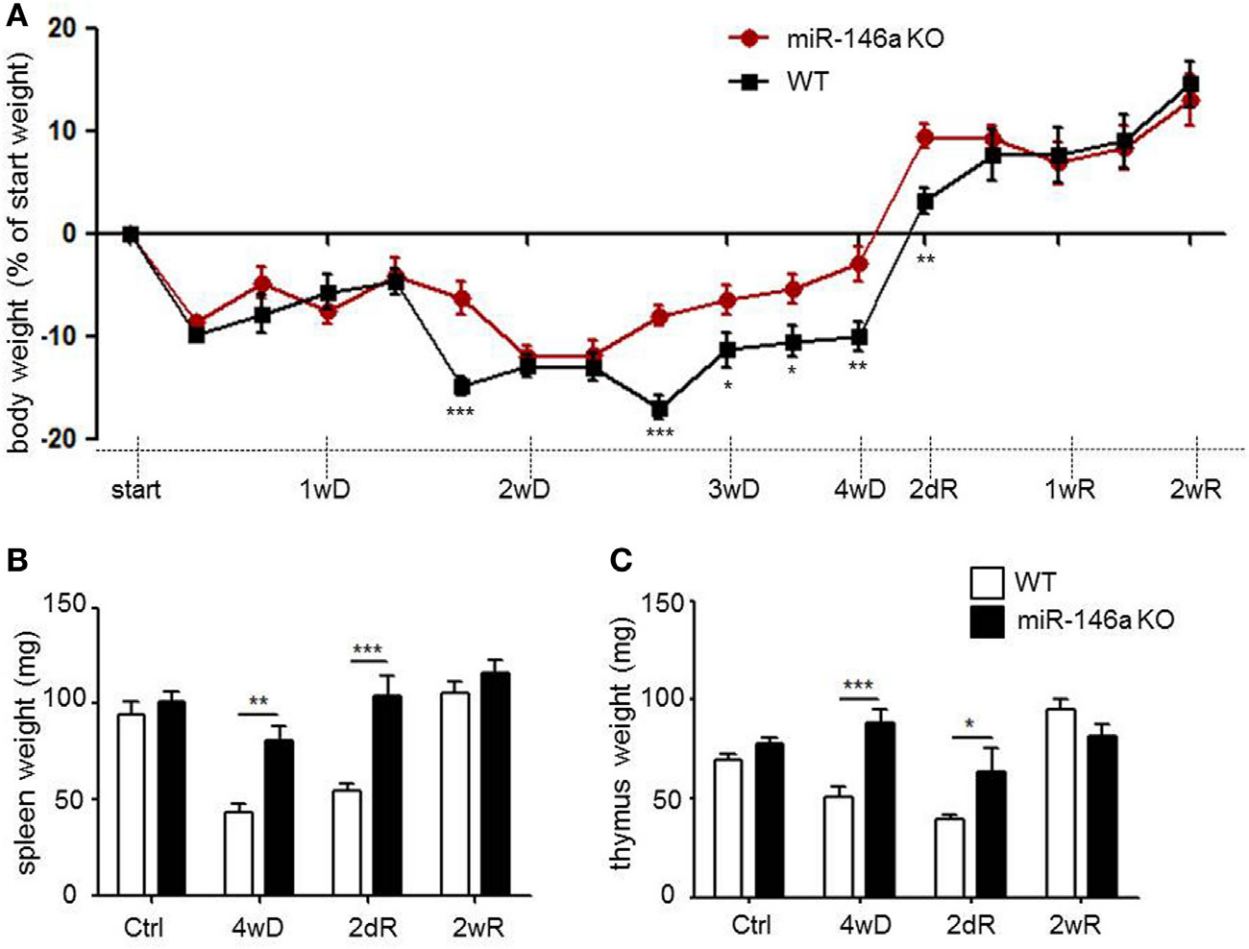
Systemic effects of cuprizone in miR-146a-deficient mice. **(A)** Body weight in response to CPZ exposure in miR-146a KO and WT mice. *p < 0.05, **p < 0.01, ***p < 0.001, *n* = 16–4 in each group at each time-point (30 mice altogether), two-way ANOVA, mean ± SEM. **(B)** Weight of thymus and **(C)** weight of spleen in response to CPZ exposure in miR-146a KO and WT mice. *p < 0.05, **p < 0.01, ***p < 0.001, *n* = 4–8 in each group, mean ± SEM. Abbreviations: 1wD: 1 week demyelination, 2wD: 2 weeks demyelination, 3wD: 3 weeks demyelination, 4wD: 4 weeks demyelination. 2dR: 2 days remyelination, 1wR: 1 week remyelination, 2wR: 2 weeks remyelination.

Weight loss is a characteristic systemic effect of CPZ exposure in mice (29). As expected, both WT and miR-146a KO mice lost weight in response to CPZ exposure, but miR-146a KO mice lost significantly less weight than WT mice during the period of demyelination (Figure 3A).

Recently, we observed thymus atrophy as an additional systemic effect of CPZ exposure (39). We, therefore, examined the effect of miR-146a deficiency on thymus weight in response to CPZ exposure. The spleen weight was also examined, since the highest expression level of miR-146a outside the CNS was found in this organ (Figure 2A). We observed atrophy of the thymus and spleen in the WT mice, whereas atrophy of both organs was less severe or absent in the miR-146a KO mice (*p* < 0.0001, spleen, and thymus, respectively) (Figures 3B,C). To determine, if this observation was an artifact of the more pronounced weight loss observed in the WT mice, we also analyzed the organ weight as a percentage of bodyweight, but these analyses showed similar results (*p* < 0.00001, spleen; *p* = 0.001, thymus, data not shown). These data indicate that miR-146a KO mice are protected against systemic effects of CPZ exposure, and this becomes evident after 2 weeks of treatment.

### Effect of miR-146a Deficiency on CPZ-Induced Demyelination and Axonal Loss

**Figure 4:**
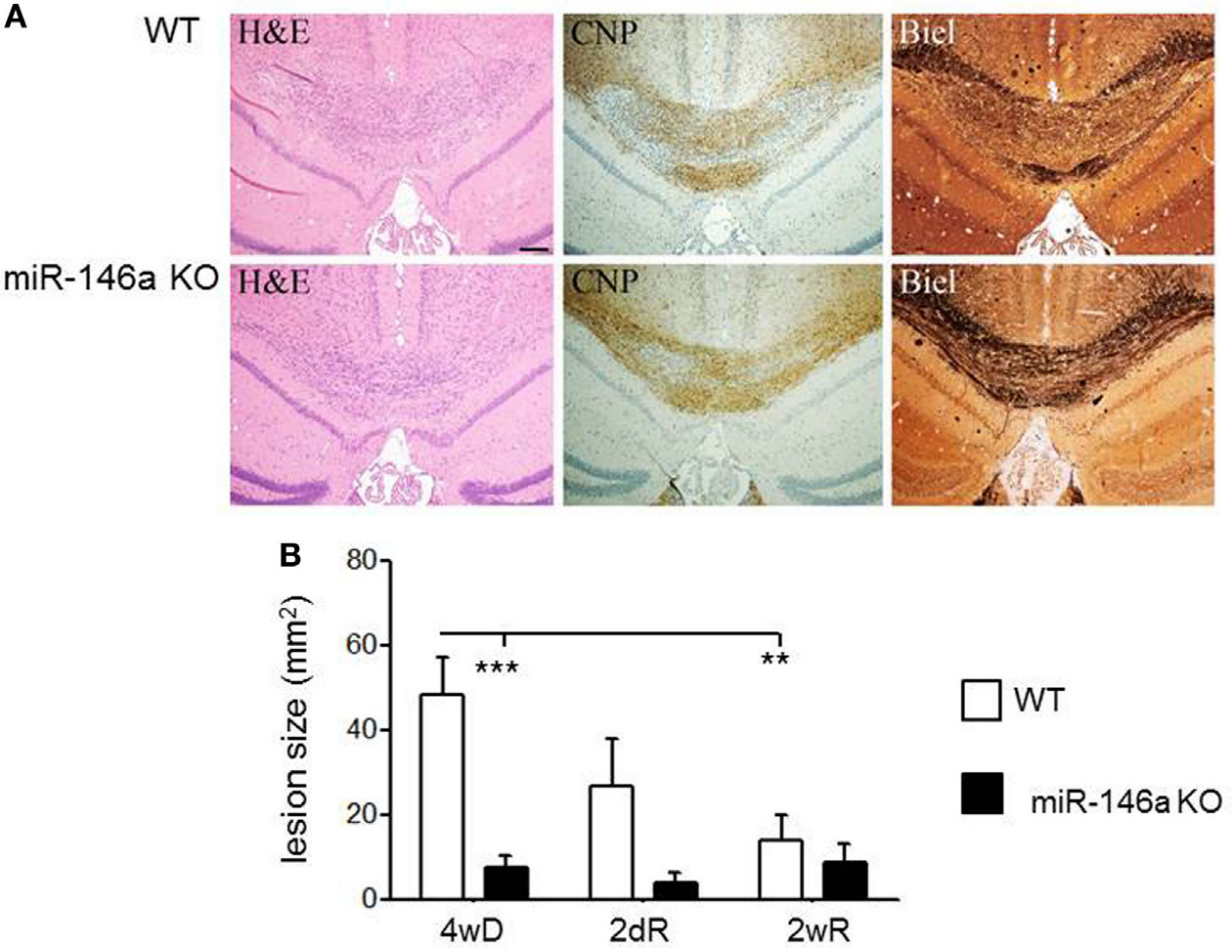
Cuprizone (CPZ)-induced demyelination and remyelination in miR-146-deficient mice. **(A)** Demyelination and axonal loss after 4 weeks of CPZ exposure in miR-146a KO and WT mice determined by Luxol fast blue (LFB) and Bielschowsky staining (Biel). **(B)** Quantification of demyelination after 4 weeks CPZ treatment (4wD) and remyelination after 2 days (2dR) and 2 weeks remyelination (2wR) in the corpus callosum of miR-146a KO and WT mice (**p < 0.01, ***p < 0.001, *n* = 4–7 in each group, two-way ANOVA, mean ± SEM).

Next, we quantified demyelination in the brain of miR-146a KO and WT mice exposed to CPZ. In the miR-146a KO mice, demyelination and axonal damage was significantly reduced (*p* = 0.0001, two-way ANOVA, Bonferroni *post hoc* test) (Figures 4A,B).

**Figure 5:**
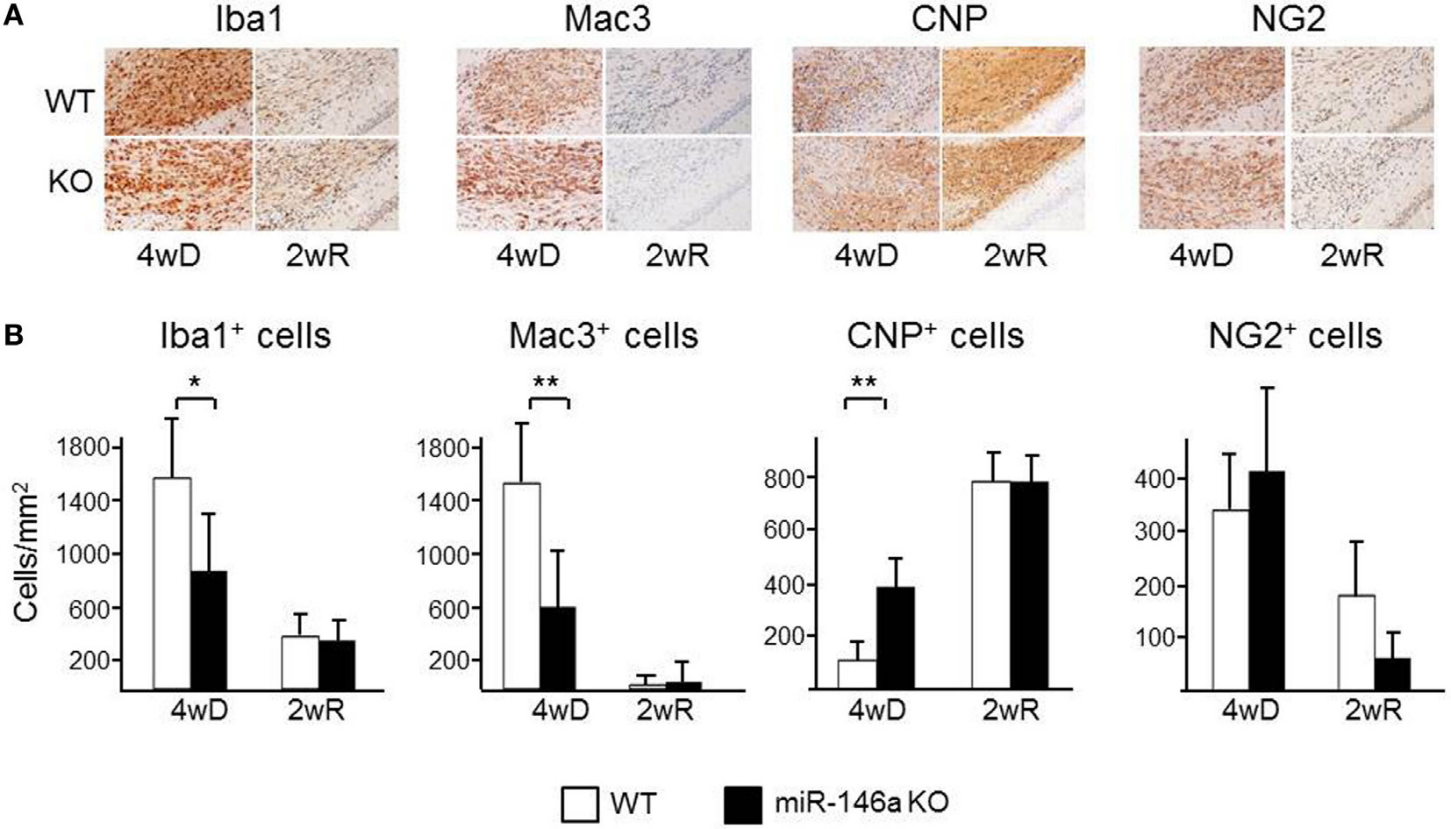
Cellular infiltration in the corpus callosum in miR-146a-deficient mice during cuprizone (CPZ)-induced demyelination and remyelination. **(A)** Myelin-producing oligodendrocytes (CNP), oligodendrocyte precursor cells (OPCs; NG2), microglia (Iba1), and macrophages (Mac3) in the corpus callosum of WT and miR-146a KO mice exposed to CPZ for 4 weeks demyelination (4wD) or exposed to CPZ for 4 weeks followed by CPZ suspension for 2 weeks remyelination (2wR). **(B)** Quantification of Mac3^+^ and Iba1^+^ macrophages/microglia, CNP^+^ myelinating oligodendrocytes and NG2^+^ OPCs in the demyelinating (4 weeks on CPZ, 4wD) and remyelinating (2 weeks on CPZ, 2wR) corpus callosum of WT and miR-146a KO mice. *p < 0.05, **p < 0.01.

The number of CNP^+^ myelinating oligodendrocytes was higher in miR-146a KO mice compared to WT mice during demyelination (*p* = 0.01) (Figure 5), while the number of NG2^+^ oligodendrocyte precursors was not different. In addition, we found a decreased number of Mac3^+^ (*p* < 0.05) (Figure 5) and a tendency of fewer Iba1^+^ cells in the corpus callosum in the KO mice during demyelination (43% less, *p* = 0.06). Two weeks after suspending CPZ, the demyelinated lesions were to a large extent remyelinated, contained Iba1^+^ cells, but only a minority of these cells was Mac3^+^. There was a modest increase of CNP^+^ cells and decrease of NG2^+^ during remyelination; no difference have been observed between KO and WT mice in CNP^+^ oligodendrocytes at this time-point, but we observed a trend of reduced number of NG2^+^ oligodendrocyte precursor cells (*p* = 0.06) (Figure 5).

### Expression of Experimentally Validated miR-146a Target Genes and Protein Products in Response to CPZ Exposure

**Figure 6:**
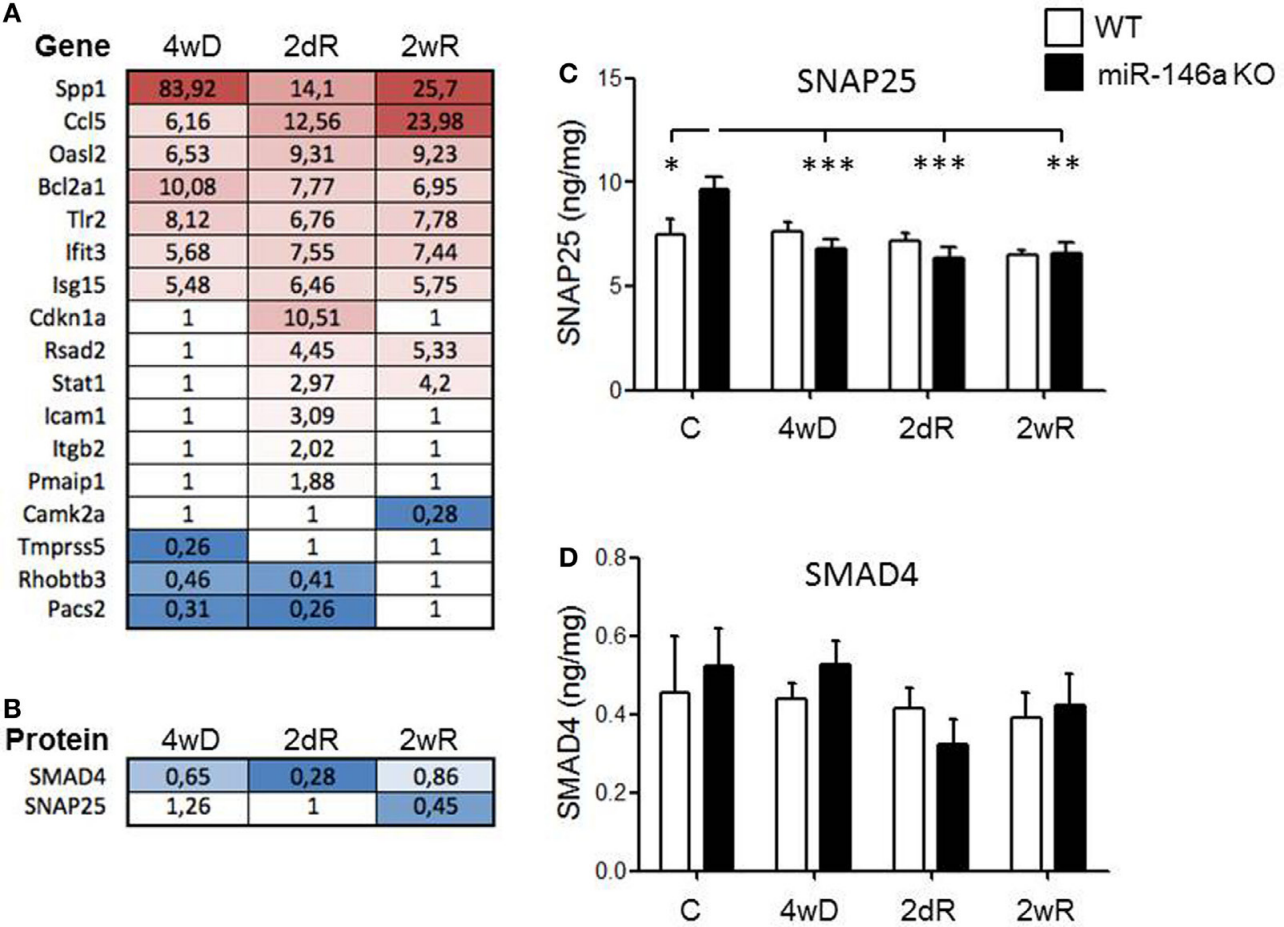
Expression of experimentally validated miR-146a target genes and proteins in response to CPZ exposure in wild-type and miR-146-deficient mice. **(A)** Expression of miR-146a target genes (miRTarBase) among 1,239 differentially expressed genes in the corpus callosum in response to CPZ exposure determined by a 4 × 44K Agilent Whole Mouse Genome Microarray (NCBI Gene Expression Omnibus with accession GSE100663). **(B)** The protein expression of validated miR-146a target genes SMAD4 and SNAP25 were downregulated in the corpus callosum of miR-146a KO mice compared to WT mice by liquid chromatography mass spectrometry in response to CPZ. **(C)** SNAP25 and **(D)** SMAD4 in corpus callosum lysates of miR-146a KO and WT mice in response to CPZ examined by ELISA. *p < 0.05, **p < 0.01, ***p < 0.001, *n* = 8–4 in each group, mean ± SEM. Abbreviations: C: un-manipulated controls, 4wD: 4 weeks demyelination. 2dR: 2 days (acute) remyelination, 2wR: 2 weeks (full) remyelination.

We extracted a list of experimentally validated miR-146a target genes from the database miRTarBase (40), and compared this list of genes with our transcriptome and proteome datasets during CPZ-induced de- and remyelination. We identified 13 upregulated and 4 downregulated miR-146a target genes among 1,239 differentially expressed genes in the corpus callosum in response to CPZ exposure determined by a 4 × 44K Agilent Whole Mouse Genome Microarray (unpublished, NCBI Gene Expression Omnibus with accession GSE100663) (Figure 6A). Based on a PANTHER GO enrichment analysis (41) of the differentially regulated miR-146a target genes, inflammatory pathways were highly enriched (Table 1). We also identified 2 downregulated protein products of miR-146a target genes in our proteome dataset (403 differentially regulated proteins, unpublished) (Figure 6B): SMAD4 (mothers against decapentaplegic homolog 4), which is known to be involved in OPC migration and differentiation (42, 43), and SNAP25 (synaptosomal-associated protein 25), which is important in the signal transduction of neurons and neurotransmitter release (44). We used ELISA assays to examine if SMAD4 and SNAP25 were differentially expressed among miR-146a KO mice and WT mice in the corpus callosum in response to CPZ exposure. As expected, we observed an increased protein level of SNAP25 in the corpus callosum of KO mice compared to WT mice in the control group. In the KO mice, the protein level of SNAP25 was reduced during de- and remyelination, while there was no change in the WT mice (*p* < 0.01, two-way ANOVA, Bonferroni *post hoc* test) (Figure 6C). The SMAD4 protein level was not significantly changed in any of the two strains of mice, and SMAD4 protein was not differentially regulated at any of the examined time-points (Figure 6D).

**Table 1:** Overrepresented biological processes of experimentally validated miR-146a target genes differentially expressed in WT mice during cuprizone-induced demyelination.

GO biological process	Fold enrichment	*p*-Value
Negative regulation of viral genome replication	93.01	0.037
Leukocyte cell–cell adhesion	90.74	0.04
Positive regulation of nitric oxygen biosynthetic process	84.55	0.049
Positive regulation of NF-kappaB transcription factor activity	45.09	0.014
Defense response to virus	36.47	0.032
Innate immune response	14.47	0.019
Intracellular signal transduction	7.85	0.022
Regulation of apoptotic process	7.28	0.039

*Pathway analysis was performed using PANTHER online software. The fold enrichment is the number of genes in our dataset representing the given biological process relative to the number of genes expected by change in a random list of genes*.

*GO, gene ontology*.

### Expression of Cytokines, Chemokines, and TNF Receptors in Response to CPZ Exposure

Based on the role of miR-146a in the regulation of inflammatory responses, and the observation that inflammatory pathways were highly enriched among the miR-146a target genes differentially regulated in response to CPZ exposure, we analyzed and compared the expression level of TNF receptor 1 (TNF-RI) and TNF receptor 2 (TNF-RII) in addition to several inflammatory cytokines and chemokines (CCL2, CCL3, CXCL1, IFN-gamma, IL-1beta, IL-2, IL-4, IL-5, IL-6, IL-10, IL-12p70, MMP-9, TNF, and VEGF) in KO versus WT mice.

**Figure 7:**
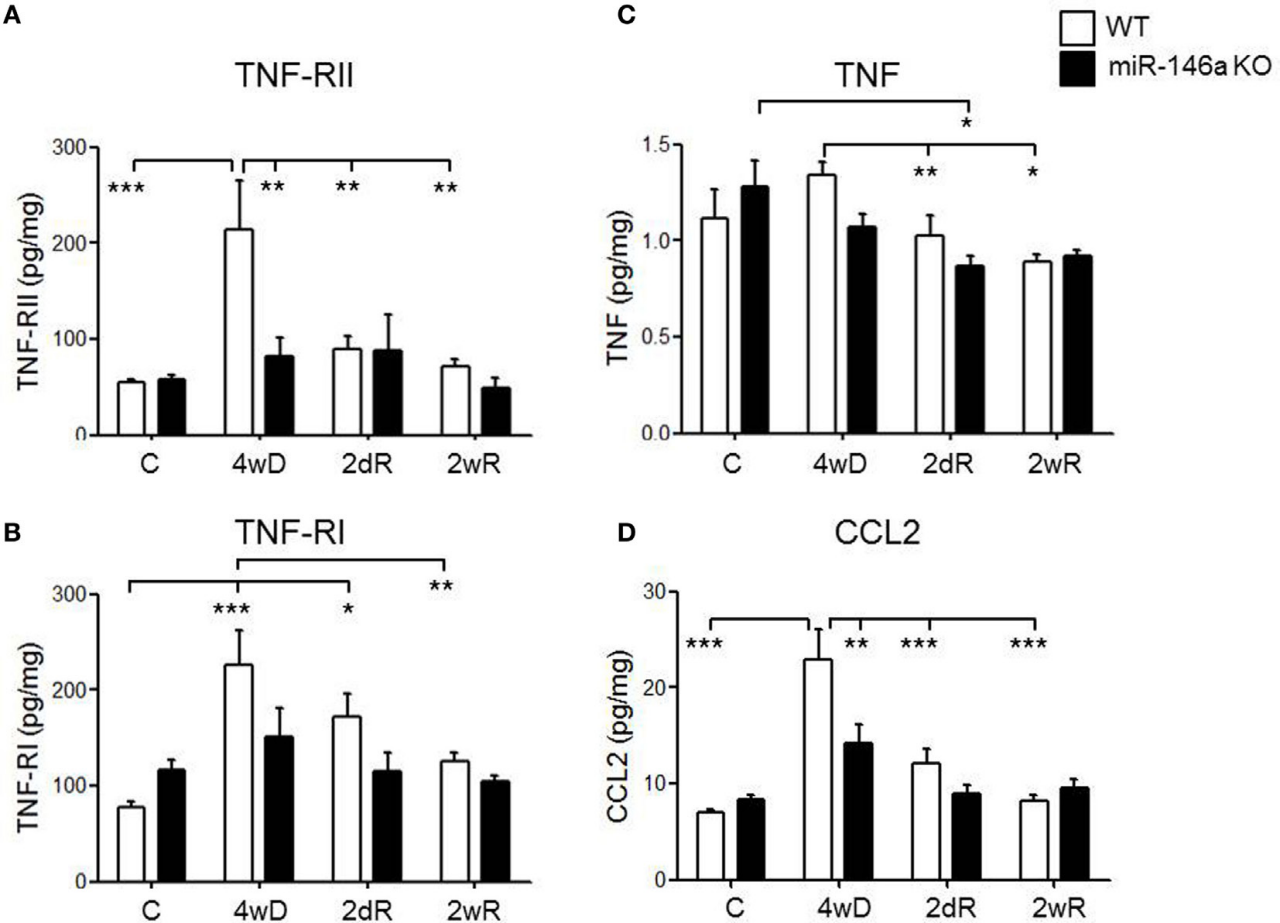
Expression of cytokines, cytokine receptors, and chemokines in the corpus callosum during experimental demyelination and remyelination in miR-146-deficient mice. Expression levels of TNF-RII **(A)**, TNF-RI **(B)**, TNF **(C)**, and CCL2 **(D)** measured by MSD-electrochemiluminescent assay in the corpus callosum of WT and miR-146a KO mice exposed to CPZ. *p < 0.05, **p < 0.01, ***p < 0.001, *n* = 8–4 in each group, mean ± SEM. Abbreviations: C, un-manipulated controls, 4wD, 4 weeks demyelination. 2dR, 2 days (acute) remyelination, 2wR, 2 weeks (full) remyelination.

Both TNF-RI and TNF-RII were significantly upregulated in the WT mice in response to CPZ exposure, but not in the miR-146a KO mice (*p* < 0.001, respectively, two-way ANOVA, Bonferroni *post hoc* test) (Figures 7A,B). In fact, during demyelination, the expression level of TNF-RII was significantly lower in the miR-146a KO mice compared to the WT mice (*p* < 0.01) (Figure 7A).

TNF expression was significantly lower during remyelination compared to demyelination in the WT mice (*p* < 0.01). In miR-146a KO mice, the highest expression of TNF was found in the control group, which was significantly higher than the expression during acute remyelination (*p* < 0.05). There was no significant difference in TNF expression level at any time-point between the two groups of mice (Figure 7C).

We also observed a significant elevation in the protein levels of chemokine CCL2 in response to CPZ exposure in the WT mice, but not in the miR-146a KO mice (*p* < 0.001, two-way ANOVA, Bonferroni *post hoc* test). In addition, the level of CCL2 was significantly lower in miR-146a KO compared to the WT mice during demyelination (*p* < 0.01) (Figure 7D).

For the additional cytokines and chemokines, we found that IL-1β was upregulated and IL-2, IL-5, IL-6, and IL-12p70 were downregulated in miR-146a KO mice, whereas IL-10 and VEGF were downregulated in both miR-146a KO mice and WT mice in response to CPZ exposure (Table 2). However, there was no significant difference in expression levels of any of these cytokines and chemokines between miR-146a KO mice and WT mice at the examined time-points (demyelination, acute, and full remyelination).

**Table 2:** Expression of cytokines and chemokines in the corpus callosum during cuprizone-induced de- and remyelination in wild-type (WT) and miR-146a KO mice.

	4wd	2dr	2wr		4wd	2dr	2wr
**IL-1beta**				**IL-12p70**			
WT	1.76	2.03	1.06	WT	0.89	0.76	0.71
miR-146aKO	1.92*	1.21	0.91	miR-146a KO	0.75	0.59*	0.70

**IL-2**				**CCL3**			
WT	0.85	0.78	0.76	WT	1.3	1.24	0.78
miR-146a KO	0.71*	0.60**	0.62*	miR-146a KO	0.82	0.65	0.64

**IL-5**				**CXCL1**			
WT	0.83	0.78	0.74	WT	1.29	1.01	0.86
miR-146a KO	0.69**	0.58***	0.60**	miR-146a KO	1.02	0.74	0.71

**IL-6**				**MMP-9**			
WT	1.02	0.82	0.79	WT	1.08	1.27	1.35
miR-146a KO	0.80	0.72*	0.74	miR-146a KO	1.03	0.73	1.39

**IL-10**				**VEGF**			
WT	0.82	0.70	0.66*	WT	0.79	0.72	0.64*
miR-146a KO	0.72*	0.63*	0.62*	miR-146a KO	0.74*	0.57**	0.64*

Results are presented relative to the control levels of the particular strain. The expression level of these cytokines and chemokines were not significantly different between WT mice and miR-146a KO mice at any of the examined time-points. *p < 0.05, **p < 0.01, ***p < 0.001, n = 8–4 in each group.

*4wd: 4 weeks demyelination. 2dr: 2 days (acute) remyelination, 2wr: 2 weeks (full) remyelination*.

## Discussion

Here, we used the CPZ mouse model of experimental de- and remyelination to mimic de- and remyelination pathology of MS (30), and examine differentially expressed miRNAs during de- and remyelination. Based on a microarray analysis followed by verification with qPCR, we identified three miRNAs, miR-146a, miR-181b, and miR-193a that were differentially expressed in response to CPZ exposure. All three miRNAs have previously been found to be differentially regulated in MS lesions: miR-146a and miR-193a were upregulated in active MS lesions, whereas miR-181b was found to be down regulated in inactive MS lesions (8). So far, four studies investigated expression and effect of miRNAs in the CPZ model, but all used different experimental setups. One study investigated the expression of miR-124 in hippocampal demyelination using dietary CPZ combined with intraperitoneal injection of rapamycin (9). In two recent studies, miR-219 recombinant retrovirus injection into the corpus callosum attenuated demyelination in the CPZ model (45), and injection of miR-146a mimics into the demyelinated corpus callosum promoted remyelination (26). Only one recent study examined miRNA expression during CPZ-induced demyelination by microarray, but miRNAs were only evaluated in sorted CNPase-EGFP^+^ cells with an OPC phenotype; only part of the differentially expressed miRNAs were presented, and those did not show miR-181b, miR-146a, or miR-193a (46).

We chose to further investigate the role of miR-146a in experimental demyelination, because (i) the expression levels of this miRNA was continuously increasing during de- and remyelination, which is in line with the finding of others (27); (ii) previous data indicated differential expression in different tissues and compartments of patients with MS, i.e., in brain lesions, body fluids, and cells (7, 8, 13, 18, 19); and (iii) miR-146a is known to be involved in regulation of the inflammatory response and survival processes of cells, including OPCs, which are relevant to MS demyelination (20–26, 32, 33).

miR-146a is highly expressed in microglia in the brain (47, 48). However, the observed increase in miR-146a in response to CPZ exposure in the corpus callosum cannot be solely explained by an increase in infiltrating microglia and macrophages, because the number of infiltrating microglia cells declines already 1 week after CPZ suspension (49, 50), and we found the highest level of miRNA-146a in the full remyelination phase, i.e., 2 weeks after suspending CPZ. Despite the increasing levels of miR-146a expression in the CNS, CPZ did not induce increased expression in a number of other organs, including the liver and the thymus, organs also affected by CPZ. This may indicate that the increased expression of miR-146a is unique to the CSN in response to CPZ-induced de- and remyelination. Therefore, we also examined the level of miR-146a in response to physiological myelination in mice at early postnatal days, but we did not observe an increase in the expression level. These data suggest that the observed increase in miR-146a levels in the corpus callosum in response to CPZ exposure is a regulated process related to pathological demyelination and remyelination.

In order to further investigate the role of miR-146a during experimental de- and remyelination, we used a miR-146a KO strain (33). We compared the systemic and CNS effects of CPZ exposure between KO and WT mice. Mice are known to lose weight in response to CPZ exposure, and we have recently recognized thymus atrophy with loss of double-positive thymocytes as an additional systemic effect of CPZ exposure (39). Here, we found that CPZ also induced atrophy of another immune organ, the spleen. Previous data suggested that administration of CPZ ameliorates EAE and delays the progressive course of Theiler’s murine encephalomyelitis (51, 52). It is possible that the additive effect of CPZ on primary and secondary immune organs may contribute to a deficiency of immune responses. We also found that miR-146a KO mice were protected against these systemic effects of CPZ: the atrophy of the thymus, spleen, and loss of body weight were all reduced in the KO mice. The most significant loss of weight was observed after 2 weeks of CPZ administration, and the protection was significant after 3 weeks. These data suggest that miRNA-146a may be involved in regulation of toxic responses and mitochondrial dysfunction, considering the mitochondrial effect of CPZ. Indeed, recent data indicate that differential expression and single nucleotide polymorphism of miR-146a can be related to drug-induced hepato- and cardiotoxicity (53, 54). In addition, miR-146a is one of the mitochondria-enriched miRNAs with potential targets on mitochondrial mRNAs, and it is most upregulated in senescent cells with mitochondrial dysfunction, altered fission, and fusion (55, 56).

Absence of miR-146a also reduced demyelination and axonal loss. The observed decrease in lesion size in miR-146a KO mice was accompanied by lower numbers of Mac3^+^ and Iba^+^ macrophages/microglia, and a higher number of CNP^+^ myelinating oligodendrocytes in the corpus callosum during demyelination. Protective effects of miR-146a deficiency have been also shown in other degenerative experimental models. In a rat model for temporal lobe epilepsy (57) and in a mouse model for Alzheimer’s disease (58), miR-146a was found to be upregulated in the brain, and inhibition by antagomiR-146a in these models led to decreased episodes of seizures and partly restored memory function, respectively. A very recent paper suggested that administration of miR-146a mimics into the corpus callosum of mice during remyelination enhanced remyelination and promoted OPC differentiation (27). In our study, miR-146 deficiency had no effect on remyelination and did not influence the number of OPCs during remyelination. Nevertheless, it reduced demyelination and axonal loss along with increased number of oligodendrocytes during demyelination. Since we did not find an increase of NG2^+^ OPCs in the KO mice during demyelination, the higher number of myelinating oligodendrocytes in the demyelinating corpus callosum may indicate increased survival of oligodendrocytes. This may also be related to change in the oligodendrocyte environment and in function of other resident cells due to the general absence of miR-146a. Interestingly, we observed a trend of reduced number of oligodendrocyte precursors in the KO mice during remyelination that may suggest that miR-146a may be beneficial during remyelination as previously suggested (27). These data may indicate a complex role of miR-146a in de- and remyelination, when examined at tissue level. The reduced axonal damage may be related to less demyelination, but axonal damage may be partly independent of demyelination in the CPZ model (59). Since mitochondrial alterations are important in axonal damage in MS, some of the protective effect in the KO mice may be related to the mitochondrial miR-146a pathways in the axons.

Next, we searched for proteins of validated target genes in our proteome database obtained during CPZ-induced de- and remyelination. We found two proteins to be downregulated during demyelination: SMAD4 and SNAP25. SMAD4 is involved in OPC migration and differentiation (42, 43), and SNAP25 is important in signal transduction of neurons and in neurotransmitter release (44). We, therefore, examined the levels of these two proteins in lysates of the corpus callosum dissected from the WT and miR-146a KO mice during de- and remyelination. The concentration of SNAP25 was increased in the miR-146a KO mice, as expected. SNAP25 was downregulated during demyelination in the miR-146a KO mice, but was not different from those in the WT mice during demyelination. Thus, ELISA results did not suggest differential regulation of SMAD4 and SNAP25 in the KO mice during demyelination.

miR-146a is a well-known negative regulator of the immune system which has been thoroughly investigated both *in vivo* and *in vitro* (21). We found that CCl2 was upregulated in WT mice in response to CPZ treatment, which is in line with results obtained by others (60). However, miR-146a KO mice expressed CCL2 in significantly lower levels during demyelination. CCL2 is a chemokine that is highly expressed by astrocytes in response to inflammatory events leading to attraction of immune cells, especially monocytes, to the inflammatory site (61). In the EAE model, conditional knockdown of CCL2 in astrocytes reduced the clinical score and infiltration by inflammatory microglia and macrophages, and delayed axonal damage in the spinal cord (62). This finding is in line with our observation that Mac3^+^ cells were reduced in the miR-146a KO mice during demyelination along with decreased levels of CCL2. In addition, we found that TNF-RI and TNF-RII levels were increased in the WT mice, but not in the miR-146a KO mice, and that there was a significant difference in the expression levels of these proteins between WT mice and miR-146a KO mice during demyelination. Previous data showed that TNF facilitated the toxic effect of CPZ on oligodendrocytes *in vitro*, and induced the depletion of microglia *in vivo*, the main source of cytokine and chemokine expression in the brain. This in turn resulted in protection against CPZ-induced demyelination (63). Therefore, it is likely that the reduction of TNF-RI, TNF-RII, and CCL2 in the miR-146a KO mice contributed to the protection against CPZ-induced demyelination. In this study, we did not examine the cellular source of these dysregulated molecules in the KO mice. The reduced levels of TNF-RI, TNF-RII, and CCL2 may reflect differential expression by different cells in the demyelinating corpus callosum, and this can influence the ultimate protective versus detrimental effect; the observed protection from CPZ-induced demyelination is a combinatory effect of miR-146a deficiency in all cell types, and we cannot exclude the possibility that lack of miR-146a in particular cell types could be harmful. We hypothesize that pro-apoptotic properties of miR-146a (23, 24, 32, 64) may also contribute to decreased demyelination, increased number of myelinating oligodendrocytes and reduced axonal loss during the demyelination phase induced by CPZ in KO mice.

In summary, here we used a comprehensive and unbiased approach to identify three miRNAs, miR-146a, miR-181b, and miR-193a, which were differentially regulated in the corpus callosum in response to CPZ exposure. We further investigated the effect of absence of miR-146a, and found that the number of oligodendrocytes was higher during demyelination in miR-146a KO mice, and demyelination and axonal loss were reduced. In addition, there was no increase of CCL2 in the demyelinating corpus callosum of the KO in contrast to the WT mice, and CCL2 levels were lower in the KO mice; this may explain the observed fewer number of infiltrating macrophages/microglia. Contrary to WT mice, there was no increase in the levels of TNF receptors in the corpus callosum of the KO mice in response to CPZ, indicating reduced inflammatory changes that may be related to the reduced number of macrophages/microglia. Altogether, these findings may suggest increased survival of oligodendrocytes, reduced production of CCL2 by astrocytes, less microglia/macrophage activation and TNF receptor expression in the KO mice, which result in reduced demyelination and axonal loss. We also observed a mild increase in NG2^+^ OPCs during remyelination in the KO mice that may support previous data indicating the beneficial role of miR-146a during remyelination (33). Additional studies should address the cellular source of the altered molecules in the corpus callosum, and if administration of antagomirs in WT mice results in similar changes.

## Ethics Statement

All animal experiments complied with the EU Directive 2010/63/EU for animal experiments. Protocols and guidelines approved by the Danish Animal Health Care Committee (2014-15-00369).

## Author Contributions

NM performed CPZ experiments, quantitative PCR, Meso Scale arrays, pathway analysis, and wrote the manuscript. VM performed microarray experiments and contributed to writing the manuscript. GS contributed to CPZ experiments and proteomics. ME, JO, AW, and ET contributed to CPZ and related experiments. AN performed proteomics and contributed to writing the manuscript. MP contributed to microdissection. FG contributed to supervising part of the CPZ experiments, proteomics, and contributed to writing the manuscript. ML supervised and designed proteomics. HL performed histology and quantification of de- and remyelination and cells. EB participated in design of the study, supervision, and contributed to writing the manuscript. TO participated in design of the study, supervision, and contributed to writing the manuscript. AS participated in design of the study, supervision, and contributed to writing the manuscript. ZI conceived the idea, designed the study, supervised, and contributed to writing the manuscript.

## Conflict of Interest Statement

The authors declare that the research was conducted in the absence of any commercial or financial relationships that could be construed as a potential conflict of interest.
